# Prevalence of Hepatitis B Vaccination Coverage and Serologic Evidence of Immunity Among US-Born Children and Adolescents From 1999 to 2016

**DOI:** 10.1001/jamanetworkopen.2020.22388

**Published:** 2020-11-11

**Authors:** Michael H. Le, Yee Hui Yeo, Samuel So, Ed Gane, Ramsey C. Cheung, Mindie H. Nguyen

**Affiliations:** 1 Division of Gastroenterology and Hepatology, Stanford University Medical Center, Palo Alto, California; 2 Asian Liver Center, Stanford University School of Medicine, Stanford, California; 3 Department of Medicine, The University of Auckland, Auckland, New Zealand; 4 Division of Gastroenterology and Hepatology, Veterans Affairs Palo Alto Health Care System, Palo Alto, California

## Abstract

**Importance:**

The World Health Assembly has called for the elimination of hepatitis B and C by 2030. As hepatitis B has no cure, the US strategy to eliminate hepatitis B has focused on prevention through vaccination. However, there are limited data on the trend in vaccine-associated immunity since the US implementation of universal infant hepatitis B vaccination.

**Objective:**

To compare self-reported hepatitis B vaccination coverage among children and adolescents with serologic evidence of immunity and infection in the US from 1999 to 2016.

**Design, Setting, and Participants:**

This population-based cross-sectional study used data from the National Health and Nutrition Examination Survey (NHANES) from 1999 to 2016. US-born persons aged 2 to 18 years without missing hepatitis B serologic test results and with reported vaccination history were included. Data were analyzed from September 2017 to June 2018.

**Main Outcomes and Measures:**

The proportion of participants who reported complete vaccination for hepatitis B and who had positive serologic test results indicating immunity.

**Results:**

Of 21 873 children and adolescents, 51.2%% were male, and the mean (SD) age was 10.6 (4.6) years. The survey reported that hepatitis B vaccination coverage increased significantly from 1999 to 2016 (from 62.6% [95% CI, 58.6%-66.4%] to 86.3% [95% CI, 82.9%-89.2%]; *P* < .001). Vaccine-associated immunity also increased from 1999 to 2016 among children aged 2 to 5 years (from 60.7% [95% CI, 48.8%-71.4%] to 65.2% [95% CI, 57.4%-72.3%]; *P* = .001) but decreased among children aged 6 to 10 years (from 64.6% [95% CI, 57.7%-70.9%] to 46.5% [95% CI, 39.1%-54.0%]; *P* < .001), adolescents aged 11 to 13 years (from 68.8% [95% CI, 58.1%-77.8%] to 26.2% [95% CI, 18.6%-35.5%]; *P* < .001), and adolescents aged 14 to 18 years (from 68.5% [95% CI, 62.9%-73.6%] to 15.6% [95% CI, 12.2%-19.8%]; *P* < .001). By birth year, serologic evidence of vaccine-associated immunity significantly decreased in the 1994-2003 NHANES birth cohort but not among those born between 1988 and 1993. Non-US–born children and adolescents did not show the same decreasing trend in immunity.

**Conclusions and Relevance:**

In this cross-sectional study, decreasing hepatitis B immunity was observed among US-born children and adolescents in the 1994-2003 NHANES birth cohort despite increasing rates of hepatitis B vaccination coverage. These findings suggest a possible need for surveillance and a booster vaccine dose for hepatitis B as those without serologic evidence of immunity become young adults and may engage in behaviors associated with an increased risk for infection.

## Key Points

**Question:**

What are the trends for self-reported hepatitis B vaccination coverage and serologic evidence of immunity in children and adolescents in the US?

**Findings:**

In this cross-sectional study of data for 21 873 US children and adolescents from National Health and Nutrition Examination Survey (NHANES) from 1999 to 2016, despite improvements in completion of the hepatitis B vaccine series, significant decreases in serologic evidence of immunity were identified in persons born in the 1994-2003 NHANES birth cohort.

**Meaning:**

The findings suggest a possible need for surveillance and a booster vaccine dose for hepatitis B for all young adults born after 1994, especially for those who engage in behaviors associated with a high risk for infection.

## Teaser

This cross-sectional study used NHANES data from 1999 to 2016 to assess the trends in self-reported hepatitis B vaccination coverage and serologic evidence of immunity among children and adolescents in the US.

## Introduction

Chronic hepatitis B (CHB) is one of the most common liver diseases, affecting approximately 257 million people worldwide.^1,2^ Although there is currently treatment to suppress the virus, there is no cure for CHB. Therefore, the global strategy for elimination is focused on prevention of hepatitis B virus (HBV) infection with universal infant HBV vaccination, which was first introduced in the US in 1991 (eFigure 1 in the Supplement). This strategy has previously been shown to be effective in countries such as Taiwan.^3^

Since the introduction of universal HBV vaccination in infants, completion of the 3-dose vaccine series has improved substantially among children aged 19 to 35 months in the US, from 68.0% in 1995 to 90.5% in 2016.^4,5^ However, the national rates of vaccination coverage were obtained through survey questionnaires and subjected to recall bias. Another approach is to confirm immunity status by serologic testing based on the assumption that reported vaccination should lead to immunity to hepatitis B.

A recent study using data from the National Health and Nutrition Examination Survey (NHANES) reported a decrease in the rates of hepatitis B vaccine–associated immunity among persons aged 6 to 19 years, from 56.8% in the 1999-2006 cohorts to 44.4% in the 2007-2012 cohorts.^6^ Whether the decrease in immunity status was associated with the uptake or durability of hepatitis B vaccines needs to be evaluated. This research is especially important because the hepatitis B vaccine preparations have changed over the years (eFigure 2 in the Supplement), which may have created a need for further studies to evaluate the effectiveness of the current combination vaccine and whether development of an improved vaccine or consideration for booster vaccination is warranted.

In addition, a waning immunity after vaccination could result in disease outbreak, as recently seen with the mumps epidemic among US university students.^7,8^ Although anamnestic response, defined by an increase in hepatitis B surface antibody (anti-HBs) titer greater than 10IU/L after a booster dose, has been shown in individuals who showed undetectable anti-HBs,^9,10^ to our knowledge, no study has compared self-reported hepatitis B vaccination coverage with vaccine-associated immunity by serologic testing in children and adolescents in the US.

The aim of this study was to use data from NHANES from 1999 to 2016 to assess the trends and rates of hepatitis B vaccination by survey history compared with evidence of persistent vaccine-associated immunity based on serologic testing in children and adolescents in the US.

## Methods

### Study Design

This cross-sectional study used data from NHANES, a national database derived from biannual reports since 1999 designed to assess the health and nutritional status of the US noninstitutionalized, civilian population.^11,12,13^ To provide national estimates, the survey uses a complex multistage, stratified, probability clustered sample design with oversampling of certain subgroups during different periods to achieve more precise evaluations. The survey was approved by the institutional review board of the US Centers for Disease Control and Prevention. Written informed consent was obtained from the participants (for those aged <18 years, consent was obtained via proxy). All data were deidentified and made publicly available.^14^ This study followed the Strengthening the Reporting of Observational Studies in Epidemiology (STROBE) reporting guideline.

### Laboratory Methods

Blood specimens were processed, stored, and shipped to the Division of Viral Hepatitis, National Center for HIV/AIDS, Viral Hepatitis, STD, and TB Prevention, Centers for Disease Control and Prevention. Enzyme-linked immunoassays were used to measure anti-HBs (AUSAB, Abbott Laboratories) and hepatitis B core antibody (anti-HBc) titers (Ortho HBc ELISA Tests System, Ortho Clinical Diagnostics) from 1999 to 2006. From 2007 onward, anti-HBc and anti-HBs titers were tested using the VITROS Reagent Packs and VITROS Immunodiagnostic Products Calibrators, respectively (Ortho Clinical Diagnostics). Results are expressed qualitatively as positive or negative for anti-HBc and anti-HBs.

Immunity to hepatitis B was defined by a positive anti-HBs test result (anti-HBs titer >10 IU/L from 1999 to 2006 and ≥12 IU/L from 2007 to 2016).^15,16^ The detection limit to define a negative anti-HBs test less than or equal to 10 IU/L from 1999 to 2006 and less than or equal to 5 IU/L from 2007 to 2016, as used by NHANES during these survey cycles.^15,16^

### Definition of Hepatitis B Vaccination Status

From the NHANES self-reported survey on HBV vaccination, complete, partial, or no vaccination were defined as reporting “yes, at least 3 doses,” “less than 3 doses,” or “no doses,” respectively. The vaccine history among those younger than 16 years or those who could not answer the questions themselves was obtained by proxy (ie, adults living in the same household).

Vaccine-associated immunity was defined as testing positive for anti-HBs and negative for anti-HBc among children and adolescents aged 6 to 18 years. In children younger than 6 years who were not tested for anti-HBc, vaccine-associated immunity was defined by anti-HBs positivity only. This definition was considered reasonable because the exposure rate to HBV infection in this age group is low.

### Statistical Analysis

Logistic regression analysis was used for trend analysis. A multivariable logistic regression model was performed to evaluate the association between various factors and HBV vaccination. Multicollinearity was assessed by variance inflation factors and condition indexes. Statistical significance was defined as 2-tailed *P* < .05. All statistical analyses were performed using Stata, version 15.1 (StataCorp). The svy suite of commands in Stata was applied using NHANES weights, which account for the complex survey design, survey nonresponse, poststratification, and oversampling and allows for the sample to be representative of the US noninstitutionalized population.

## Results

### Study Participants

**Figure 1.**
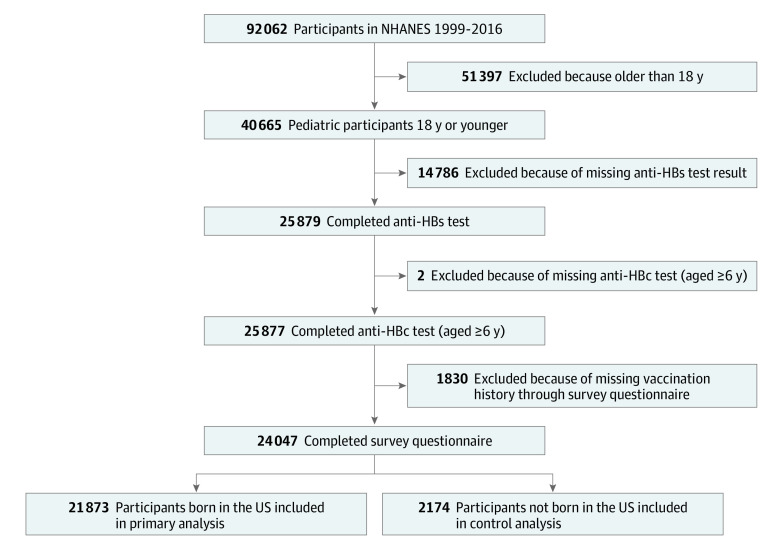
Flowchart of Pediatric Participants in National Health and Nutrition Examination Survey (NHANES) From 1999 to 2016 anti-HBc indicates hepatitis B core antibody; and anti-HBs, hepatitis B surface antibody.

Between 1999 and 2016, a total of 40 664 children and adolescents aged 2 to 18 years were enrolled in NHANES, of whom 24 047 completed HBV serologic tests as well as survey questionnaires (Figure 1). Because the policy and practice of infant hepatitis B vaccination likely differ between the US and other countries, only the 21 873 US-born children and adolescents (51.2% male; mean [SD] age, 10.6 [4.3] years) were included in the analysis of the primary study group. The 2169 non-US–born children and adolescents were analyzed separately for comparison.

### Hepatitis B Vaccination Status by Survey Questionnaire and Serologic Evidence of Immunity From 1999 to 2016

**Figure 2.**
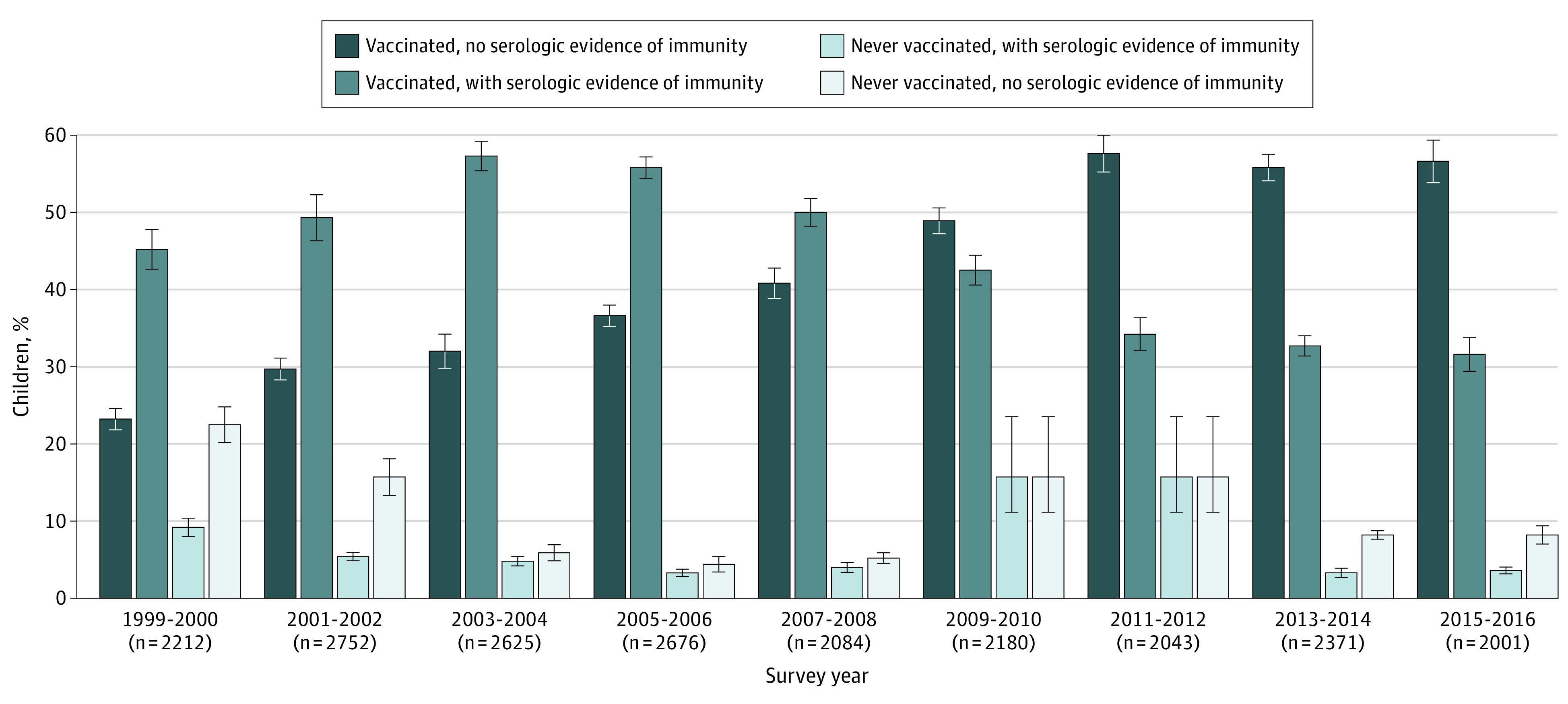
Reported Hepatitis B Vaccination Status and Serologic Evidence of Immunity Among US Children and Adolescents Aged 2 to 18 Years From 1999 to 2016 All *P* < .001 for trend for all 4 subgroups. Error bars indicate linearized SE.

Survey-based complete hepatitis B vaccination rates significantly increased over time (from 62.6% [95% CI, 58.6%-66.4%] in the 1999-2000 cohort to 86.3% [95% CI, 82.9%-89.2%] in the 2015-2016 cohort; *P* < .001) (Figure 2). Vaccine-associated immunity rates based on serologic testing among those with reported complete vaccination also increased from 41.4% (95% CI, 36.6%-46.2%) in the 1999-2000 cohort to 54.3% (95% CI, 50.9%-57.5%) in the 2003-2004 cohort (*P* < .001) but decreased steadily after 2004 (from 54.3% [95% CI, 50.9%-57.5%] in the 2003-2004 cohort to 30.9% [95% CI, 27.0%-35.1%] in the 2015-2016 cohort; *P* < .001). A total of 13.0% (95% CI, 10.5%-16.0%) surveyed in the 1999-2000 NHANES and 4.5% (95% CI, 3.5%-5.7%) surveyed in the 2015-2016 NHANES who reported receiving no doses of the hepatitis B vaccine also had evidence of vaccine-associated immunity based on serologic testing.

### Hepatitis B Vaccination and Vaccine-Associated Immunity by Age Group

**Figure 3.**
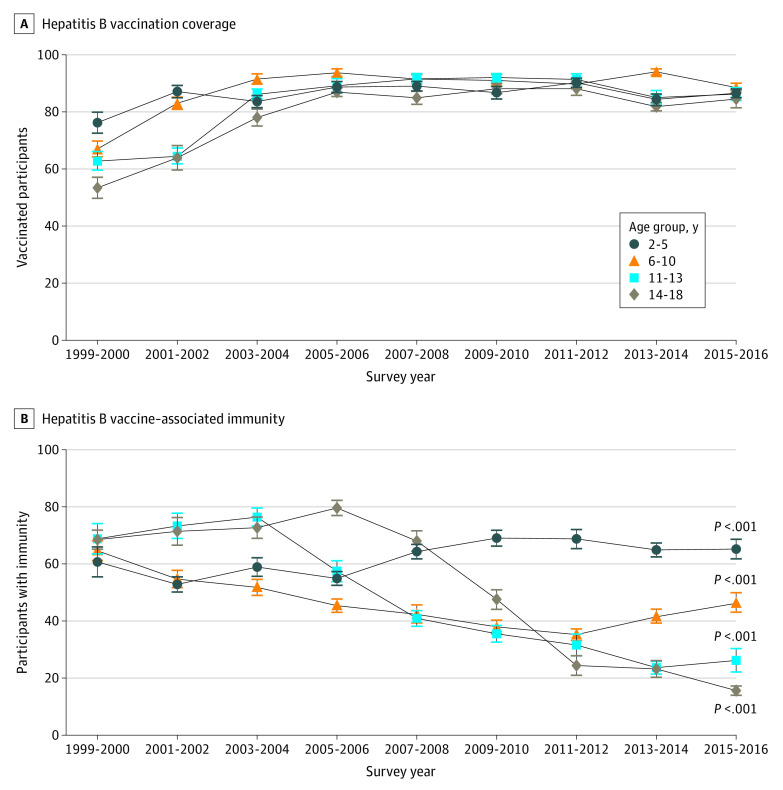
Hepatitis B Vaccination Coverage and Vaccine-Associated Immunity by Age Group Among US Children and Adolescents Aged 2 to 18 Years From 1999 to 2016 Error bars indicate linearized standard error. *P* values are from the test for trend within each age group.

From 1999 to 2006, there was an increase in reported hepatitis B vaccine series completion among children and adolescents consistently across all age groups, reaching 81.8% (95% CI, 78.9%-84.3%) or greater from 2005-2006 to 2015-2016 (Figure 3A), and across race/ethnicity, sex, and socioeconomic subgroups (eTable 1 in the Supplement). Similar increases were observed among non-US–born children and adolescents (eTable 2 in the Supplement).

The trend in vaccine-associated immunity based on serologic testing varied among the different age groups (Figure 3B). From 1999 to 2016, vaccine-associated immunity among children aged 2 to 5 years increased from 60.7% (95% CI, 48.8%-71.4%) to 65.2% (95% CI, 57.4%-72.3%) (*P* = .001) but decreased among children aged 6 to 10 years from 64.6% (95% CI, 57.7%-70.9%) to 46.5% (95% CI, 39.1%-54.0%) (*P* < .001). Among adolescents aged 11 to 13 years, vaccine-associated immunity increased from 68.8% (95% CI, 58.1%-77.8%) in the 1999-2000 cohort to 76.4% (95% CI, 68.7%-82.6%) in the 2003-2004 cohort but decreased steadily to 26.2% (95% CI, 18.6%-35.5%) in the 2015-2016 cohort (*P* < .001). Similarly, vaccine-associated immunity among adolescents aged 14 to 18 years increased from 68.5% (95% CI, 62.9%-73.6%) in the 1999-2000 cohort to 79.6% (95% CI, 74.2%-84.1%) in the 2005-2006 cohort (*P* = .004) but decreased steadily to 15.6% (95% CI, 12.2%-19.8%) in the 2015-2016 cohort (*P* < .001). This decrease in vaccine-associated immunity in adolescents aged 11 to 18 years was only observed among those born in the US but not among non-US–born children and adolescents or across various demographic characteristics, including sex, race/ethnicity, education level, income level, household size, and health insurance status (eTable 3 and 4 in the Supplement). However, the prevalence of positive anti-HBc test results decreased to 0.15% (95% CI, 0.09%-0.27%) in the 2011-2016 cohorts from 0.53% (95% CI, 0.31%-0.93%) in the 2005-2010 cohorts (eTable 5 in the Supplement).Among those who were positive for anti-HBc (n = 96), 68.4% (95% CI, 48.2%-83.4%) reported having completed the hepatitis B vaccine series. Among those who were positive for anti-HBs and reported no history of hepatitis B vaccination (n = 1005), 11 were found to be anti-HBc positive (1.2% [95% CI, 0.3%-4.9%]). On multivariable logistic regression, older age and non-Hispanic Asian ethnicity were associated with a significantly lower odds of having detectable anti-HBs (eTable 6 in the Supplement).

### Vaccine-Associated Immunity Among Those With Complete Vaccination by Age Group and Birth Year

**Figure 4.**
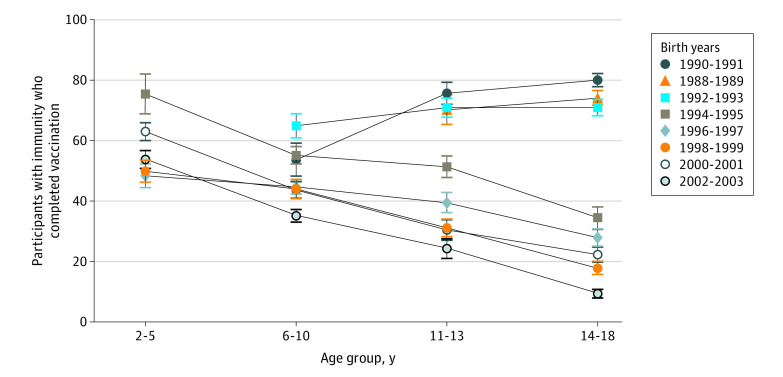
Vaccine-Associated Immunity in Children and Adolescents Aged 2 to 18 Years With Reported Complete Hepatitis B Vaccination by Birth Year (1988-2003) and Age Group in the US Error bars indicate linearized SE.

Among children and adolescents who reported completing the hepatitis B vaccine series, higher rates of vaccine-associated immunity were found in children aged 2 to 5 years compared with older children (Figure 4). In the 1988-1993 birth cohorts, the rate of vaccine-associated immunity increased with increasing age. However, in the 1994-2003 birth cohorts, the rate of vaccine-associated immunity decreased steadily with age. In addition, we observed a significant difference in prevalence of anti-HBs in persons born before 1999 vs after 1999 (54.5% [95% CI, 52.4%-56.6%] vs 45.5% [95% CI, 43.6%-47.4%]; *P* < .001). Self-reported vaccination rates increased steadily from 1988 to 1993 and then stabilized and remained high among those born later (eTable 7 in the Supplement). After adjusting for age, race/ethnicity, health insurance status, and income level, we observed that younger age, having health insurance, higher income level, and Mexican-American and Hispanic ethnicity were independently associated with completing the hepatitis B vaccine series (eTable 8 in the Supplement).

## Discussion

To our knowledge, this is the first study to examine in detail the trend in reported hepatitis B vaccination coverage and vaccine-associated immunity based on serologic testing among children and adolescents in the US. To our knowledge, this is also the first nationwide, population-based study to assess trends in detectable vaccine-associated immunity by NHANES birth cohorts among children and adolescents born in the US between 1994 and 2003.

In this study of NHANES data, we found increasing hepatitis B vaccination coverage by participant self-report, which is in agreement with prior reports by the National Immunization Survey conducted by the US Centers for Disease Control and Prevention which obtained data directly from vaccination providers.^4,17,18^ Despite this finding, in birth cohorts between 1994 and 2003 (the last birth year with data for the 14- to 18-year age group), we observed a consistent decrease in vaccine-associated immunity. This decrease occurred despite significant increases in hepatitis B vaccine series completion among children aged 19 to 35 months, with more than 80% of children completing the vaccine series from 1996 to 1999 and approximately 90% of children completing the series from 2000 onward.^4,5,19,20^ Among US-born children and adolescents who reported complete hepatitis B vaccination, hepatitis B immunity in adolescents aged 14 to 18 years was low (52.5%) overall and was lowest in the 2002-2003 birth cohort (9.2%). Of note, a similar decrease was not observed among non-US–born children and adolescents (eTable 4 in the Supplement). The only group with a sustained or increased vaccine-associated immunity based in serologic testing was that of youths aged 6 to 18 years who were born between 1988 and 1993. The introduction of policies aimed at catch-up vaccination in 1999 and 2005 may partly explain the higher rates of vaccine-associated immunity in this group (eFigure 1 in the Supplement). However, whether there are other factors that were associated with the sustained increase in vaccine-associated immunity is unclear because we could not determine the rate of immunization through catch-up vaccination in this population.

For participants who were born between 1994 and 2003, there are a few potential explanations for the observed decrease in vaccine-associated immunity despite increased rates of self-reported vaccination. First, the decrease may be attributable to recall bias from the participants or their parents, such that the actual rates of vaccination were lower. However, recall bias is unlikely to be the primary cause of the discrepancy between survey and serologic results observed in this study because the reported vaccination coverage was consistent with data directly obtained from vaccine providers by the National Immunization Survey.^21^

Second, the decrease in vaccine-associated immunity may have been associated with anti-HBs levels waning over time. Prior studies with up to 30-year-follow-up, including a meta-analysis, showed that anti-HBs level could wane over time.^22,23,24,25,26^ However, we do not believe our observations in this study are attributable to waning antibody levels alone. If the decrease in vaccine-associated immunity was attributable to waning levels of anti-HBs, we would have likely seen similar rates of vaccine-associated immunity among participants in the same age group across all birth cohorts. Instead, we observed significant decreases in vaccine-associated immunity in certain (1994-1995 and 2002-2003) but not all birth cohorts.

Third, the removal of thimerosal hepatitis B vaccines in 1999 may have changed the immunogenicity of the vaccines and served as a potential cause for this phenomenon. A study^27^ found no significant differences in antibody level decreases or distribution between persons who received thimerosal-containing and thimerosal-free vaccines, but this study only included adults aged 18 to 50 years and there were no significant differences in the slope of decrease among persons who were born before and after 1999. In addition, infants were only vaccinated with the monovalent hepatitis B vaccine manufactured before 1996, and whether the decrease in immunity seen in this study^27^ was associated with the later adoption of combination vaccines to complete the vaccine series is unclear. In the current study, we observed a significant association of vaccination with prevalence of anti-HBs before 1999 vs after 1999. However, it is unclear whether this decrease was attributable to changes in vaccines alone. In addition, there may be a differential effect of vaccination in infants compared with adolescents owing to immune maturity. A prior meta-analysis^28^ on hepatitis B response in young adults found that those who were vaccinated at a younger age had higher seroprotection rates than did those vaccinated at an older age, suggesting that vaccination may be more effective in younger individuals.

Fourth, the rates of vaccine-associated immunity based on serologic test results would be lower if the sensitivity of the anti-HBs test used in NHANES was lower in the latter period. However, this was not the case because the detection limit for the anti-HBs test used in NHANES has actually been lowered to 5 IU/mL (previously 10 IU/mL) since 2007.^15,16^

Fifth, the trend of decreasing vaccine-associated immunity was not observed among non-US–born children and adolescents. This finding could be attributable to current vaccination requirements recommended by the US Centers for Disease Control and Prevention^29^; in which, immigrants to the US are required to provide proof of prior hepatitis B vaccination. Some of these children and adolescents may also have received a different HBV vaccine preparation that may be more immunogenic in their countries of origin. In addition, non-US–born children may have a higher prevalence of vaccine-associated immunity because they may have been vaccinated at an older age before immigration to the US.

Of note, although universal infant vaccination was initiated in Taiwan in 1984, decreasing hepatitis B immunity as measured by anti-HBs levels has also been observed among university students (aged ≥18 years) in Taiwan who were born in 2009 (36.4%) compared with those born in 1984 (73.9%).^30,31,32^ The lack of detectable anti-HBs does not automatically confer lack of immunity because prior studies^9,10,33,34,35,36,37,38^ have also shown that the majority of these previously vaccinated children and adolescents would have an anamnestic response on reexposure to HBV (eAppendix in the Supplement). In a study with 22 years of follow-up, McMahon et al^34^ reported that 195 of 493 Alaskan Natives (40%) had an anti-HBs level less than 10 mIU/mL. Anamnestic response was shown in 81% and 41% of the participants 60 days and 1 year after they received a booster dose, respectively. However, the study used plasma-derived hepatitis B vaccine, which was discontinued in 1990. In addition, the birth cohort included in that study included individuals born in the early 1980s, before the birth cohorts that showed decreases in immunity based on serologic test results in our study (between 1994 and 2003). Therefore, the sustainability of vaccinations administered after 1993 might not be comparable to that in the study by McMahon et al.^34^ The birth years of study participants in prior studies of vaccine anamnestic response are in the 1980s (eAppendix in the Supplement). In 2 of these studies,^9,33^ vaccines were administered in 1992 and 2001 but the study samples of these 2 studies were small (20 and 40, respectively), limiting their conclusions. In another long-term study^39^ of combined hepatitis A and B vaccine in adults, immunity to HBV remained detectable in 89.3% to 92.9% of participants after 15 years, and an anamnestic response was found in all 4 patients who received a booster dose.

According to the World Health Organization guideline for HBV vaccination, there is no evidence to recommend a booster dose to those who completed the vaccine series and have a low risk of infection.^40^ However, it is recommended that people at high risk of infection receive a booster dose if their anti-HBs level is less than 10 mIU/mL.^40,41^ Our study demonstrated a significant decrease in vaccine-associated immunity in the later years of the study period, suggesting the importance of reexamining the need for surveillance. In addition, there might be need for a booster dose in selected populations, such as teenagers engaged in high-risk activities. In fact, some researchers have suggested that individuals aged 13 to 19 years be closely monitored to determine whether a booster dose is needed if their anti-HBs level becomes undetectable.^41^ The incidence of acute hepatitis B infection among children and adolescents aged 0 to 19 years has remained low in the US from 2000 to 2015.^42^ In addition, despite the waning immunity, serologic evidence of hepatitis B infection in children and adolescents has remained minimal. This finding could be attributable to herd immunity given that the universal HBV vaccination program in the US was launched in 1991. In addition, the lack of measurable anti-HBs level despite completion of the hepatitis B vaccine series does not necessarily indicate a lack of immunity. Several studies^9,10,33,34,35,36,37,38^ on immune memory have found that a majority of individuals who are no longer positive for anti-HBs many years after vaccination can still mount an immune response after receiving a hepatitis B booster dose (eAppendix in the Supplement). Nevertheless, future studies should be considered to determine the mechanism for the decreasing immunity in the observed birth cohorts and the associated clinical implication regarding future needs for booster vaccination.

### Strengths and Limitations

This study has strengths. It provides an extension and an update to previous reports on HBV vaccination coverage by including the most recent years of data from NHANES (2013-2016) with more representation from Asian participants (first sampled in 2011).^13^ Use of NHANES data allowed us to objectively evaluate evidence of vaccination and immunity via serologic test results, whereas previous studies^17,18^ assessed the rates of vaccination only by contacting vaccine providers for patient vaccine history. The complex sampling design of NHANES also minimizes the likelihood for selection bias, and by weighting the data during the analysis, we were able to produce accurate data for the general US population. In addition, we excluded non-US–born children and adolescents to avert the bias caused by the heterogeneity of vaccination policies in different countries. We also focused only on children and adolescents, who might have a different immune response than adults, especially when the vaccine series is started at birth, whereas most of the other long-term immunity studies focus on adults.

This study also has limitations. We used detectable anti-HBs as an indicator for immunity; however, as discussed above, anti-HBs levels can wane with time and the absence of the antibody does not always indicate a lack of immunity.^43^ Prior studies^9,10,33,34,35,36,37,38^ have shown that a persistent immune memory response remains among a majority of those who no longer have detectable anti-HBs many years after vaccination, as evidenced by emergence of protective anti-HBs levels after reexposure with a hepatitis B booster dose (eAppendix in the Supplement). The scope of coverage of NHANES is also restricted to the noninstitutionalized population in the US, and these data cannot be generalized to high-risk groups, such as incarcerated, institutionalized, and homeless individuals.

## Conclusions

This cross-sectional study revealed increasing hepatitis B vaccination coverage in the US from 1999 to 2016 overall and across most subgroups, with narrower gaps between some of the subgroups with greater disparities. However, there was a significant decrease in the rates of vaccine-associated immunity, as measured by anti-HBs titers, in individuals born between 1994 and 2003, suggesting a possible need to assess the effectiveness of the hepatitis B vaccine and protection from HBV infection. The findings also suggest the need for surveillance and a possible booster dose for individuals with undetectable levels of anti-HBs when they become young adults and may be at increased risk for infection through unprotected sex or injection drug use.

## Notes

Supplement. **eTable 1.** Prevalence of complete hepatitis B vaccination in U.S.-born children and adolescents (2-18 years) through self-reported vaccination history (survey years: 1999-2016) **eTable 2.** Prevalence of complete hepatitis B vaccination in foreign-born children and adolescents (2-18 years) through self-reported vaccination history (survey years: 1999-2016) **eTable 3.** Prevalence of Hepatitis B vaccine-induced immunity by serology results in fully vaccinated U.S.-born children and adolescents age 2-18 years (survey years: 1999-2016) **eTable 4.** Prevalence of complete hepatitis B vaccine-induced immunity by serology test in fully vaccinated foreign-born children and adolescents age 2-18 years (survey years: 1999-2016) **eTable 5.** Prevalence of hepatitis B core antibody in U.S.-born children and adolescents age 2-18 years (survey years 1999-2016) **eTable 6.** Predictors of hepatitis B immunity via serology tests in fully vaccinated U.S.-born children (2-18 years) from survey years 1999-2016 **eTable 7.** Age specific prevalence of complete vaccination through participant self-reported vaccination history, by birth year from 1988-2007 **eTable 8.** Predictors of hepatitis B vaccination through reported vaccine history in U.S.-born children and adolescents (2-18 years) from survey years 1999-2016 **eFigure 1.** Timeline of hepatitis B vaccine recommendations by the Advisory Committee on Immunization Practices **eFigure 2.** Timeline of hepatitis B vaccine availability in the United States **eAppendix.** Literature review on anamnestic response **eReferences**
