# Supplementary material for: Prevalence of Hepatitis B Vaccination Coverage and Serologic Evidence of Immunity Among US-Born Children and Adolescents From 1999 to 2016
Source: JAMA Netw Open. 2020 Nov 11;3(11):e2022388. doi: 10.1001/jamanetworkopen.2020.22388 (PMC7658733; doi:10.1001/jamanetworkopen.2020.22388)
Supplement: Supplement. — eTable 1. Prevalence of complete hepatitis B vaccination in U.S.-born children and adolescents (2-18 years) through self-reported vaccination history (survey years: 1999-2016) eTable 2. Prevalence of complete hepatitis B vaccination in foreign-born children and adolescents (2-18 years) through self-reported vaccination history (survey years: 1999-2016) eTable 3. Prevalence of Hepatitis B vaccine-induced immunity by serology results in fully vaccinated U.S.-born children and adolescents age 2-18 years (survey years: 1999-2016) eTable 4. Prevalence of complete hepatitis B vaccine-induced immunity by serology test in fully vaccinated foreign-born children and adolescents age 2-18 years (survey years: 1999-2016) eTable 5. Prevalence of hepatitis B core antibody in U.S.-born children and adolescents age 2-18 years (survey years 1999-2016) eTable 6. Predictors of hepatitis B immunity via serology tests in fully vaccinated U.S.-born children (2-18 years) from survey years 1999-2016 eTable 7. Age specific prevalence of complete vaccination through participant self-reported vaccination history, by birth year from 1988-2007 eTable 8. Predictors of hepatitis B vaccination through reported vaccine history in U.S.-born children and adolescents (2-18 years) from survey years 1999-2016 eFigure 1. Timeline of hepatitis B vaccine recommendations by the Advisory Committee on Immunization Practices eFigure 2. Timeline of hepatitis B vaccine availability in the United States eAppendix. Literature review on anamnestic response eReferences [file jamanetwopen-e2022388-s001.pdf]

# Supplemental Online Content

Le MH, Yeo YH, So S, Gane E, Cheung RC, Nguyen MH. Prevalence of hepatitis B vaccination coverage and serologic evidence of immunity among US-born children and adolescents from 1999 to 2016. *JAMA Netw Open*. 2020;3(11):e2022388. doi:10.1001/jamanetworkopen.2020.22388

**eTable 1.** Prevalence of complete hepatitis B vaccination in U.S.-born children and adolescents (2-18 years) through self-reported vaccination history (survey years: 1999-2016)

**eTable 2.** Prevalence of complete hepatitis B vaccination in foreign-born children and adolescents (2-18 years) through self-reported vaccination history (survey years: 1999-2016)

**eTable 3.** Prevalence of Hepatitis B vaccine-induced immunity by serology results in fully vaccinated U.S.-born children and adolescents age 2-18 years (survey years: 1999-2016)

**eTable 4.** Prevalence of complete hepatitis B vaccine-induced immunity by serology test in fully vaccinated foreign-born children and adolescents age 2-18 years (survey years: 1999-2016)

**eTable 5.** Prevalence of hepatitis B core antibody in U.S.-born children and adolescents age 2-18 years (survey years 1999-2016)

**eTable 6.** Predictors of hepatitis B immunity via serology tests in fully vaccinated U.S.-born children (2-18 years) from survey years 1999-2016

**eTable 7.** Age specific prevalence of complete vaccination through participant self-reported vaccination history, by birth year from 1988-2007

**eTable 8.** Predictors of hepatitis B vaccination through reported vaccine history in U.S.-born children and adolescents (2-18 years) from survey years 1999-2016

**eFigure 1.** Timeline of hepatitis B vaccine recommendations by the Advisory Committee on Immunization Practices

© 2020 Le MH et al. *JAMA Network Open.*

**eFigure 2.** Timeline of hepatitis B vaccine availability in the United States

**eAppendix.** Literature review on anamnestic response

**eReferences**

This supplemental material has been provided by the authors to give readers additional information about their work.

© 2020 Le MH et al. *JAMA Network Open.*

**eTable 1: Prevalence of complete hepatitis B vaccination in U.S.-born children and adolescents (2-18 years) through self-reported vaccination history (survey years: 1999-2016)**

| | 1999-2004 | | 2005-2010 | | 2011-2016 | | Trends Analysis |
|---|---|---|---|---|---|---|---|
| **Demographic Variables** | **n** | **Adjusted % (95% CI)** | **n** | **Adjusted % (95% CI)** | **n** | **Adjusted % (95% CI)** | ***P*-value** |
| **Overall** | 8158 | **74.5** (72.0-76.9) | 7128 | **89.3** (87.8-90.5) | 6587 | **87.4** (85.9-88.8) | **<0.001** |
| **Sex** | | | | | | | |
| Male | 4097 | **75.8** (72.7-78.7) | 3657 | **88.7** (86.9-90.3) | 3376 | **87.6** (85.9-89.2) | **<0.001** |
| Female | 4061 | **73.2** (70.8-75.4) | 3471 | **89.8** (88.0-91.4) | 3211 | **87.2** (85.2-89.0) | **<0.001** |
| **Age groups (years)** | | | | | | | |
| 2-5 | 1288 | **83.5** (80.6-86.1) | 1561 | **88.1** (85.6-90.1) | 1493 | **86.8** (84.6-88.8) | 0.073 |
| 6-10 | 1931 | **81.1** (78.4-83.5) | 2069 | **92.1** (90.0-93.9) | 2201 | **90.8** (89.0-92.4) | **<0.001** |
| 11-13 | 1816 | **71.3** (68.1-74.4) | 1313 | **90.9** (88.4-92.9) | 1194 | **87.5** (84.8-89.8) | **<0.001** |
| 14-18 | 3123 | **65.8** (61.2-70.2) | 2185 | **86.6** (84.3-88.6) | 1699 | **84.7** (81.8-87.2) | **<0.001** |
| **Race/Ethnicity** | | | | | | | |
| Non-Hispanic White | 2330 | **74.3** (70.7-77.6) | 2239 | **88.7** (86.5-90.6) | 1773 | **87.5** (85.3-89.4) | **<0.001** |
| Non-Hispanic Black | 2805 | **72.3** (68.9-75.4) | 1945 | **87.0** (83.8-89.6) | 1787 | **85.5** (81.2-89.0) | **<0.001** |
| Mexican American | 2423 | **76.1** (71.9-79.9) | 1955 | **91.5** (89.5-93.2) | 1419 | **88.2** (85.8-90.3) | **<0.001** |
| Hispanic | 296 | **82.2** (75.6-87.3) | 594 | **93.3** (89.8-95.6) | 722 | **86.7** (83.2-89.5) | **0.268** |
| Non-Hispanic Asian | 0 | | 0 | | 451 | **87.9** (83.5-91.3) | |

© 2020 Le MH et al. *JAMA Network Open.*

| | | | | | | | |
|---|---|---|---|---|---|---|---|
| Other-Multiracial | 304 | **73.1** (63.3-81.0) | 395 | **91.5** (86.8-94.6) | 435 | **90.2** (85.8-93.4) | **<0.001** |
| **Education Level (Age ≥6)** | | | | | | | |
| ≤Elementary School | 2624 | **77.1** (74.7-79.3) | 2643 | **92.4** (90.6-93.9) | 2737 | **89.7** (87.7-91.3) | **<0.001** |
| Middle School | 2086 | **75.4** (71.6-78.7) | 1336 | **88.6** (85.9-90.9) | 1159 | **87.7** (85.1-90.0) | **<0.001** |
| ≥ High School | 2156 | **62.9** (58.0-67.6) | 1587 | **86.0** (83.4-88.3) | 1195 | **84.1** (80.7-87.0) | **<0.001** |
| **Insurance Status** | | | | | | | |
| Insured | 6935 | **75.7** (73.1-78.2) | 6358 | **90.1** (88.7-91.3) | 6149 | **88.0** (86.5-89.4) | **<0.001** |
| Uninsured | 1116 | **64.7** (58.9-70.1) | 751 | **81.6** (76.6-85.7) | 430 | **79.0** (72.4-84.3) | **<0.001** |
| **Household Size** | | | | | | | |
| 1-2 | 454 | **67.3** (59.3-74.3) | 356 | **82.9** (77.9-87.0) | 251 | **88.0** (81.0-92.6) | **<0.001** |
| 3-5 | 5495 | **76.0** (73.4-78.4) | 4852 | **89.8** (88.5-91.0) | 4348 | **87.9** (85.7-89.7) | **<0.001** |
| 6+ | 2209 | **71.1** (65.1-76.4) | 1920 | **88.7** (84.5-91.8) | 1988 | **86.1** (83.4-88.5) | **<0.001** |
| **Family poverty income ratio (PIR)** | | | | | | | |
| <1 | 2466 | **72.8** (69.3-76.0) | 2099 | **88.6** (86.0-90.7) | 2109 | **86.0** (83.3-88.3) | **<0.001** |
| ≥1 | 5163 | **75.8** (73.3-78.2) | 4650 | **89.6** (87.9-91.1) | 4034 | **88.2** (86.4-89.7) | **<0.001** |

© 2020 Le MH et al. *JAMA Network Open.*

**eTable 2: Prevalence of complete hepatitis B vaccination in foreign-born children and adolescents (2-18 years) through self-reported vaccination history (survey years: 1999-2016)**

| | 1999-2004 | | 2005-2010 | | 2011-2016 | | Trends Analysis |
|---|---|---|---|---|---|---|---|
| **Demographic Variables** | **n** | **Adjusted % (95% CI)** | **n** | **Adjusted % (95% CI)** | **n** | **Adjusted % (95% CI)** | ***P*-value** |
| **Overall** | 954 | **67.4** (61.9-72.5) | 661 | **89.0** (85.1-92.0) | 554 | **85.4** (82.0-88.3) | **<0.001** |
| **Sex** | | | | | | | |
| Male | 506 | **65.1** (57.4-72.1) | 328 | **87.1** (81.7-91.1) | 270 | **87.2** (82.2-91.0) | **<0.001** |
| Female | 448 | **69.9** (63.7-75.5) | 333 | **90.9** (86.1-94.1) | 284 | **83.6** (77.8-88.1) | **0.001** |
| **Age groups (years)** | | | | | | | |
| 2-5 | 49 | **71.2** (53.2-84.4) | 42 | **92.9** (78.6-97.9) | 50 | **91.9** (81.4-96.7) | **0.019** |
| 6-10 | 152 | **78.9** (69.8-85.9) | 155 | **90.7** (81.7-95.5) | 123 | **88.7** (81.0-93.5) | **0.042** |
| 11-13 | 195 | **67.2** (57.3-75.8) | 137 | **90.5** (80.6-95.6) | 110 | **84.4** (74.3-91.0) | **0.008** |
| 14-18 | 558 | **61.1** (53.4-68.2) | 327 | **86.8** (80.3-91.4) | 271 | **83.5** (79.0-87.2) | **<0.001** |
| **Race/Ethnicity** | | | | | | | |
| Non-Hispanic White | 66 | **73.7** (60.3-83.8) | 62 | **95.2** (84.1-98.7) | 22 | **92.4** (71.3-98.4) | **0.035** |
| Non-Hispanic Black | 109 | **68.6** (53.9-80.2) | 55 | **91.9** (78.7-97.2) | 59 | **83.2** (71.3-90.8) | 0.085 |
| Mexican American | 647 | **65.7** (59.3-71.5) | 372 | **84.1** (79.2-88.0) | 164 | **82.0** (73.8-88.1) | **0.002** |
| Hispanic | 87 | **65.2** (50.5-77.5) | 116 | **92.7** (83.7-96.9) | 106 | **91.7** (83.6-96.0) | **<0.001** |
| Non-Hispanic Asian | 0 | | 0 | | 182 | **81.7** (72.5-88.4) | |

© 2020 Le MH et al. *JAMA Network Open.*

| | | | | | | | |
|---|---|---|---|---|---|---|---|
| Other-Multiracial | 45 | **63.6** (41.4-81.2) | 56 | **85.4** (68.5-94.0) | 21 | **87.9** (64.7-96.6) | 0.056 |
| **Education Level (Age ≥6)** | | | | | | | |
| ≤Elementary School | 210 | **67.4 (61.9-72.5)** | 218 | **89.0 (85.1-92.0)** | 168 | **85.4 (82.0-88.3)** | **<0.001** |
| Middle School | 334 | **69.4 (61.1-76.6)** | 182 | **88.0 (78.6-93.6)** | 149 | **89.6 (81.1-94.5)** | **<0.001** |
| ≥ High School | 360 | **62.6** (52.6-71.5) | 219 | **86.4** (79.0-91.4) | 187 | **81.7** (75.4-86.7) | **0.001** |
| **Insurance Status** | | | | | | | |
| Insured | 435 | **70.1** (61.8-77.2) | 342 | **91.0** (86.1-94.3) | 378 | **87.3** (83.8-90.1) | **<0.001** |
| Uninsured | 511 | **64.2** (57.2-70.6) | 314 | **85.6** (79.4-90.1) | 175 | **81.7** (74.4-87.2) | **<0.001** |
| **Household Size** | | | | | | | |
| 1-2 | 26 | **52.9** (28.8-75.8) | 22 | **81.4** (54.3-94.2) | 12 | **72.9** (45.6-89.6) | 0.237 |
| 3-5 | 501 | **67.6** (58.3-75.7) | 373 | **90.2** (85.4-93.6) | 381 | **86.1** (81.8-89.6) | **<0.001** |
| 6+ | 427 | **68.5** (60.0-75.9) | 266 | **87.5** (81.0-92.0) | 161 | **85.1** (78.6-89.8) | **0.001** |
| **Family poverty income ratio (PIR)** | | | | | | | |
| <1 | 435 | **69.6** (61.7-76.4) | 300 | **88.0** (82.7-91.9) | 203 | **83.8** (75.8-89.6) | **0.005** |
| ≥1 | 423 | **66.6** (55.5-76.1) | 297 | **88.9** (83.4-92.7) | 281 | **85.7** (80.7-89.6) | **<0.001** |

© 2020 Le MH et al. *JAMA Network Open.*

**eTable 3. Prevalence of Hepatitis B vaccine-induced immunity by serology results in fully vaccinated U.S.-born children and adolescents age 2-18 years (survey years: 1999-2016)**

| | 1999-2004 | | 2005-2010 | | 2011-2016 | | Trends Analysis |
|---|---|---|---|---|---|---|---|
| **Demographic Variables** | **n** | **Adjusted % (95% CI)** | **n** | **Adjusted % (95% CI)** | **n** | **Adjusted % (95% CI)** | ***P*-value** |
| **Overall** | 7589 | **57.2** (53.8-60.6) | 6940 | **53.3** (51.2-55.4) | 6415 | **35.9** (33.7-38.2) | **<0.001** |
| **Sex** | | | | | | | |
| Male | 3815 | **55.9** (51.9-59.8) | 3554 | **52.3** (50.0-54.9) | 3288 | **35.9** (33.4-38.5) | **<0.001** |
| Female | 3774 | **58.7** (54.9-62.3) | 3386 | **54.4** (51.5-57.2) | 3127 | **35.9** (33.0-39.0) | **<0.001** |
| **Age groups (years)** | | | | | | | |
| 2-5 | 1175 | **57.2** (53.1-61.2) | 1468 | **61.7** (58.6-64.6) | 1408 | **65.9** (62.2-69.3) | **0.002** |
| 6-10 | 1856 | **53.1** (49.6-56.5) | 2043 | **41.9** (38.9-45.0) | 2162 | **40.4** (37.4-43.5) | **<0.001** |
| 11-13 | 1653 | **64.8** (59.8-69.5) | 1294 | **43.4** (39.7-47.2) | 1175 | **26.5** (22.9-30.5) | **<0.001** |
| 14-18 | 2905 | **56.8** (51.8-61.6) | 2135 | **64.0** (60.1-67.7) | 1670 | **21.2** (18.2-24.6) | **<0.001** |
| **Race/Ethnicity** | | | | | | | |
| Non-Hispanic White | 2171 | **56.5** (52.3-60.6) | 2172 | **53.0** (49.8-56.2) | 1728 | **31.9** (29.0-35.0) | **<0.001** |
| Non-Hispanic Black | 2624 | **55.5** (51.1-59.7) | 1894 | **55.6** (52.3-58.8) | 1750 | **41.7** (37.9-45.5) | **<0.001** |
| Mexican American | 2234 | **57.8** (52.6-62.8) | 1902 | **54.0** (50.8-57.1) | 1377 | **40.4** (37.2-43.6) | **<0.001** |
| Hispanic | 278 | **71.0** (62.4-78.3) | 585 | **52.4** (47.6-57.2) | 701 | **43.9** (39.9-47.9) | **<0.001** |
| Non-Hispanic Asian | 0 | | 0 | | 435 | **41.8** (37.2-46.5) | |
| Other-Multiracial | 282 | **56.1** (46.0-65.7) | 387 | **50.3** (43.7-56.9) | 424 | **34.3** (29.1-40.0) | **<0.001** |

© 2020 Le MH et al. *JAMA Network Open.*

| | | | | | | | |
|---|---|---|---|---|---|---|---|
| **Education Level (Age≥6)** | | | | | | | |
| ≤Elementary School | 2472 | **54.3** (50.8-57.7) | 2611 | **41.8** (39.1-44.6) | 2688 | **38.6** (35.9-41.4) | **<0.001** |
| Middle School | 1928 | **66.5** (61.7-70.9) | 1311 | **47.8** (42.6-53.0) | 1143 | **21.3** (17.8-25.3) | **<0.001** |
| ≥ High School | 2010 | **54.2** (48.5-59.9) | 1549 | **68.7** (64.5-72.6) | 1173 | **21.8** (18.5-25.6) | **<0.001** |
| **Insurance Status** | | | | | | | |
| Insured | 6454 | **58.1** (54.6-61.5) | 6202 | **53.3** (51.1-55.4) | 5998 | **36.1** (33.8-38.5) | **<0.001** |
| Uninsured | 1032 | **51.0** (45.2-56.7) | 720 | **53.4** (49.2-57.5) | 409 | **32.8** (26.7-39.6) | **<0.001** |
| **Household Size** | | | | | | | |
| 1-2 | 419 | **53.1** (46.1-60.0) | 343 | **58.1** (49.7-66.0) | 245 | **38.4** (29.8-47.7) | **0.020** |
| 3-5 | 5112 | **58.8** (55.5-62.1) | 4723 | **53.4** (51.0-55.8) | 4237 | **35.7** (33.1-38.3) | **<0.001** |
| 6+ | 2058 | **52.3** (46.2-58.3) | 1874 | **51.9** (49.0-54.7) | 1933 | **36.3** (32.8-39.9) | **<0.001** |
| **Family poverty income ratio (PIR)** | | | | | | | |
| <1 | 2258 | **52.9** (48.4-57.4) | 2042 | **51.3** (47.3-55.3) | 2041 | **38.4** (36.0-40.9) | **<0.001** |
| ≥1 | 4842 | **58.8** (55.1-62.4) | 4528 | **53.7** (51.1-56.3) | 3938 | **34.9** (32.3-37.6) | **<0.001** |

© 2020 Le MH et al. *JAMA Network Open.*

**eTable 4: Prevalence of complete hepatitis B vaccine-induced immunity by serology test in fully vaccinated foreign-born children and adolescents age 2-18 years (survey years: 1999-2016)**

| | 1999-2004 | | 2005-2010 | | 2011-2016 | | Trends Analysis |
|---|---|---|---|---|---|---|---|
| **Demographic Variables** | **n** | **Adjusted % (95% CI)** | **n** | **Adjusted % (95% CI)** | **n** | **Adjusted % (95% CI)** | ***P*-value** |
| **Overall** | 874 | **63.4** (58.2-68.4) | 648 | **69.5** (65.1-73.6) | 528 | **62.6** (57.3-67.7) | 0.877 |
| **Sex** | | | | | | | |
| Male | 469 | **59.9** (54.3-65.2) | 321 | **64.9** (56.9-72.2) | 259 | **60.4** (54.8-65.7) | 0.857 |
| Female | 405 | **67.2** (60.4-73.2) | 327 | **74.2** (69.4-78.4) | 269 | **65.0** (57.4-71.9) | 0.695 |
| **Age groups (years)** | | | | | | | |
| 2-5 | 41 | **78.4** (59.0-90.1) | 42 | **54.9** (37.0-71.5) | 45 | **77.5** (60.6-88.5) | 0.937 |
| 6-10 | 140 | **76.8** (67.9-83.9) | 152 | **68.1** (60.1-75.2) | 120 | **63.7** (52.7-73.4) | **0.047** |
| 11-13 | 175 | **67.8** (54.7-78.9) | 135 | **70.2** (59.1-79.4) | 103 | **65.3** (56.2-73.3) | 0.757 |
| 14-18 | 518 | **52.6** (45.3-59.8) | 319 | **72.4** (65.3-78.6) | 260 | **58.7** (49.8-67.0) | 0.272 |
| **Race/Ethnicity** | | | | | | | |
| Non-Hispanic White | 64 | **69.7** (56.0-80.6) | 61 | **70.7** (58.9-80.2) | 22 | **69.3** (42.6-87.2) | 0.996 |
| Non-Hispanic Black | 101 | **51.4** (41.1-61.7) | 53 | **51.7** (38.6-64.7) | 56 | **73.7** (60.1-83.8) | **0.013** |
| Mexican American | 590 | **60.1** (53.0-66.8) | 363 | **68.5** (62.5-73.9) | 157 | **66.9** (59.5-73.4) | 0.159 |
| Hispanic | 78 | **70.3** (59.7-79.1) | 115 | **71.3** (59.0-81.1) | 101 | **55.0** (42.1-67.3) | 0.072 |
| Non-Hispanic Asian | 0 | | 0 | | 173 | **56.6** (47.9-64.9) | |
| Other-Multiracial | 41 | **56.7** (37.4-74.2) | 56 | **73.6** (51.5-88.0) | 19 | **61.0** (28.6-85.9) | 0.440 |

© 2020 Le MH et al. *JAMA Network Open.*

| | | | | | | | |
|---|---|---|---|---|---|---|---|
| **Education Level (Age ≥6)** | | | | | | | |
| ≤Elementary School | 190 | **72.5** (63.7-79.8) | 215 | **69.3** (61.3-76.3) | 160 | **63.6** (55.2-71.3) | 0.141 |
| Middle School | 307 | **61.5** (52.7-69.6) | 175 | **68.7** (59.2-76.8) | 144 | **57.8** (47.8-67.3) | 0.564 |
| ≥ High School | 335 | **53.3** (42.5-63.8) | 216 | **74.2** (66.3-80.8) | 179 | **62.5** (53.1-71.0) | 0.172 |
| **Insurance Status** | | | | | | | |
| Insured | 403 | **67.0** (60.2-73.2) | 335 | **66.6** (60.1-72.6) | 360 | **62.3** (55.6-68.6) | 0.317 |
| Uninsured | 463 | **57.8** (50.9-64.4) | 308 | **74.5** (67.7-80.3) | 167 | **63.6** (54.9-71.5) | 0.201 |
| **Household Size** | | | | | | | |
| 1-2 | 24 | **51.1** (24.9-76.7) | 22 | **91.3** (74.5-97.4) | 11 | **84.5** (38.0-98.0) | 0.170 |
| 3-5 | 460 | **63.1** (56.2-69.5) | 366 | **67.2** (61.2-72.7) | 361 | **62.9** (56.5-68.9) | 0.964 |
| 6+ | 390 | **65.0** (56.5-72.6) | 260 | **71.5** (62.9-78.7) | 156 | **59.6** (48.1-70.2) | 0.514 |
| **Family poverty income ratio (PIR)** | | | | | | | |
| <1 | 392 | **69.9** (63.1-75.9) | 295 | **76.2** (68.4-82.6) | 191 | **63.2** (56.2-69.7) | 0.199 |
| ≥1 | 390 | **60.2** (51.8-67.9) | 289 | **66.0** (60.0-71.6) | 269 | **63.1** (55.3-70.2) | 0.598 |

© 2020 Le MH et al. *JAMA Network Open.*

**eTable 5: Prevalence of hepatitis B core antibody in U.S.-born children and adolescents age 2-18 years (survey years 1999-2016)**

| | 1999-2004 | 2005-2010 | 2011-2016 | Trends Analysis |
|---|---|---|---|---|
| **Age groups (years)** | **Adjusted % (95% CI)** | **Adjusted % (95% CI)** | **Adjusted % (95% CI)** | ***P*-value** |
| **Overall (n=17,531)** | **0.35** (0.14-0.90) | **0.53** (0.31-0.93) | **0.15** (0.09-0.27) | **0.190** |
| **Age groups** | | | | |
| **2-5** | NA | NA | NA | |
| **6-10 (n=6201)** | **0.55** (0.11-2.66) | **0.33** (0.08-1.42) | **0.15** (0.06-0.41) | **0.259** |
| **11-13 (n=4323)** | **0.21** (0.07-0.66) | **0.65** (0.24-1.74) | **0.18** (0.06-0.49) | **0.835** |
| **14-18 (n=7007)** | **0.26** (0.12-0.58) | **0.64** (0.37-1.12) | **0.14** (0.05-0.41) | **0.326** |

© 2020 Le MH et al. *JAMA Network Open.*

**eTable 6. Predictors of hepatitis B immunity via serology tests in fully vaccinated U.S.-born children (2-18 years) from survey years 1999-2016**

| Variables | Univariable Analysis | | Multivariable Analysis | |
|---|---|---|---|---|
| | Odds Ratio (95% CI) | *P*-value | Odds Ratio (95% CI) | *P*-value |
| **Age groups (years)** | | | | |
| 2-5 | 1 (Referent) | | 1 (Referent) | |
| 6-10 | **0.51** (0.46-0.56) | **<0.001** | **0.51** (0.46-0.56) | **<0.001** |
| 11-13 | **0.49** (0.43-0.56) | **<0.001** | **0.49** (0.43-0.57) | **<0.001** |
| 14-18 | **0.55** (0.48-0.63) | **<0.001** | **0.55** (0.49-0.63) | **<0.001** |
| **Sex** | | | | |
| Male | 1 (Referent) | | 1 (Referent) | |
| Female | **1.06** (0.98-1.15) | 0.133 | 1.06 (0.98-1.15) | 0.131 |
| **Race** | | | | |
| Non-Hispanic White | 1 (Referent) | | 1 (Referent) | |
| Non-Hispanic Black | **1.15** (1.02-1.31) | **0.032** | **1.14** (1.00-1.30) | **0.045** |
| Mexican American | 1.09 (0.96-1.23) | 0.193 | 1.05 (0.93-1.19) | 0.424 |
| Hispanic | **1.30** (1.08-1.55) | **0.006** | **1.27** (1.05-1.54) | **0.015** |
| Non-Hispanic Asian | **0.79** (0.65-0.97) | **0.022** | **0.79** (0.65-0.96) | **0.017** |
| Other-Multiracial | 0.99 (0.83-1.18) | 0.791 | 0.95 (0.79-1.14) | 0.566 |

© 2020 Le MH et al. *JAMA Network Open.*

**eTable 7: Age specific prevalence of complete vaccination through participant self-reported vaccination history, by birth year from 1988-2007**

| | Birth year | | | | | | | | | | |
|---|---|---|---|---|---|---|---|---|---|---|---|
| | **88-89** | **90-91** | **92-93** | **94-95** | **96-97** | **98-99** | **00-01** | **02-03** | **04-05** | **06-07** | ***P*-value** |
| **Age groups (years)** | | | | | | | | | | | |
| **2-5** | | | | **79.6** (55.9-92.3) | **84.9** (78.0-90.0) | **85.3** (81.0-88.8) | **84.9** (81.0-88.1) | **86.2** (81.9-89.7) | **89.4** (85.3-92.5) | **88.9** (84.6-92.0) | **0.031** |
| **6-10** | | **58.3** (50.6-65.6) | **74.8** (69.6-79.3) | **85.8** (82.2-88.8) | **92.6** (89.1-95.1) | **91.6** (87.9-94.3) | **92.2** (89.3-94.3) | **89.2** (84.5-92.6) | **91.9** (88.9-94.1) | **91.4** (88.0-93.8) | **<0.001** |
| **11-13** | **65.4** (59.1-71.2) | **70.6** (65.9-74.8) | **82.8** (77.6-87.0) | **91.5** (86.4-94.8) | **93.5** (90.8-95.4) | **92.8** (89.6-95.1) | **88.3** (82.9-92.2) | **84.9** (79.2-89.2) | **87.2** (83.3-90.3) | | **<0.001** |
| **14-18** | **80.0** (75.6-83.8) | **84.0** (80.3-87.1) | **86.9** (83.0-90.1) | **87.4** (84.3-90.0) | **88.7** (84.7-91.8) | **80.2** (75.5-84.2) | **85.3** (78.6-90.1) | **89.5** (78.8-95.2) | | | 0.283 |

© 2020 Le MH et al. *JAMA Network Open.*

**eTable 8. Predictors of hepatitis B vaccination through reported vaccine history in U.S.-born children and adolescents (2-18 years) from survey years 1999-2016**

| Variables | Univariable Analysis | | Multivariable Analysis | |
|---|---|---|---|---|
| | Odds Ratio (95% CI) | *P*-value | Odds Ratio (95% CI) | *P*-value |
| **Age groups (years)** | | | | |
| 2-5 | 1 (Referent) | | 1 (Referent) | |
| 6-10 | **1.16** (1.00-1.34) | **0.043** | **1.18** (1.01-1.37) | **0.037** |
| 11-13 | **0.78** (0.66-0.92) | **0.004** | **0.77** (0.64-0.91) | **0.003** |
| 14-18 | **0.61** (0.52-0.71) | **<0.001** | **0.63** (0.54-0.74) | **<0.001** |
| **Race** | | | | |
| Non-Hispanic White | 1 (Referent) | | 1 (Referent) | |
| Non-Hispanic Black | 0.88 (0.74-1.04) | 0.136 | 0.90 (0.75-1.08) | 0.272 |
| Mexican American | **1.23** (1.01-1.50) | **0.035** | **1.31** (1.08-1.58) | **0.006** |
| Hispanic | **1.38** (1.07-1.79) | **0.014** | **1.38** (1.07-1.79) | **0.015** |
| Non-Hispanic Asian | **1.46** (1.00-2.11) | **0.048** | 1.34 (0.92-1.95) | 0.129 |
| Other-Multiracial | 1.19 (0.88-1.62) | 0.257 | 1.18 (0.89-1.57) | 0.241 |
| **Insurance status** | | | | |
| Uninsured | 1 (Referent) | | 1 (Referent) | |
| Insured | **1.97** (1.65-2.36) | **<0.001** | **1.94** (1.63-2.29) | **<0.001** |
| **Family poverty income ratio (PIR)** | | | | |
| <1 | 1 (Referent) | | 1 (Referent) | |
| ≥1 | **1.16** (1.03-1.32) | **0.017** | **1.22** (1.07-1.38) | **0.003** |

© 2020 Le MH et al. *JAMA Network Open.*

**eFigure 1. Timeline of hepatitis B vaccine recommendations by the Advisory Committee on Immunization Practices**[1-12]

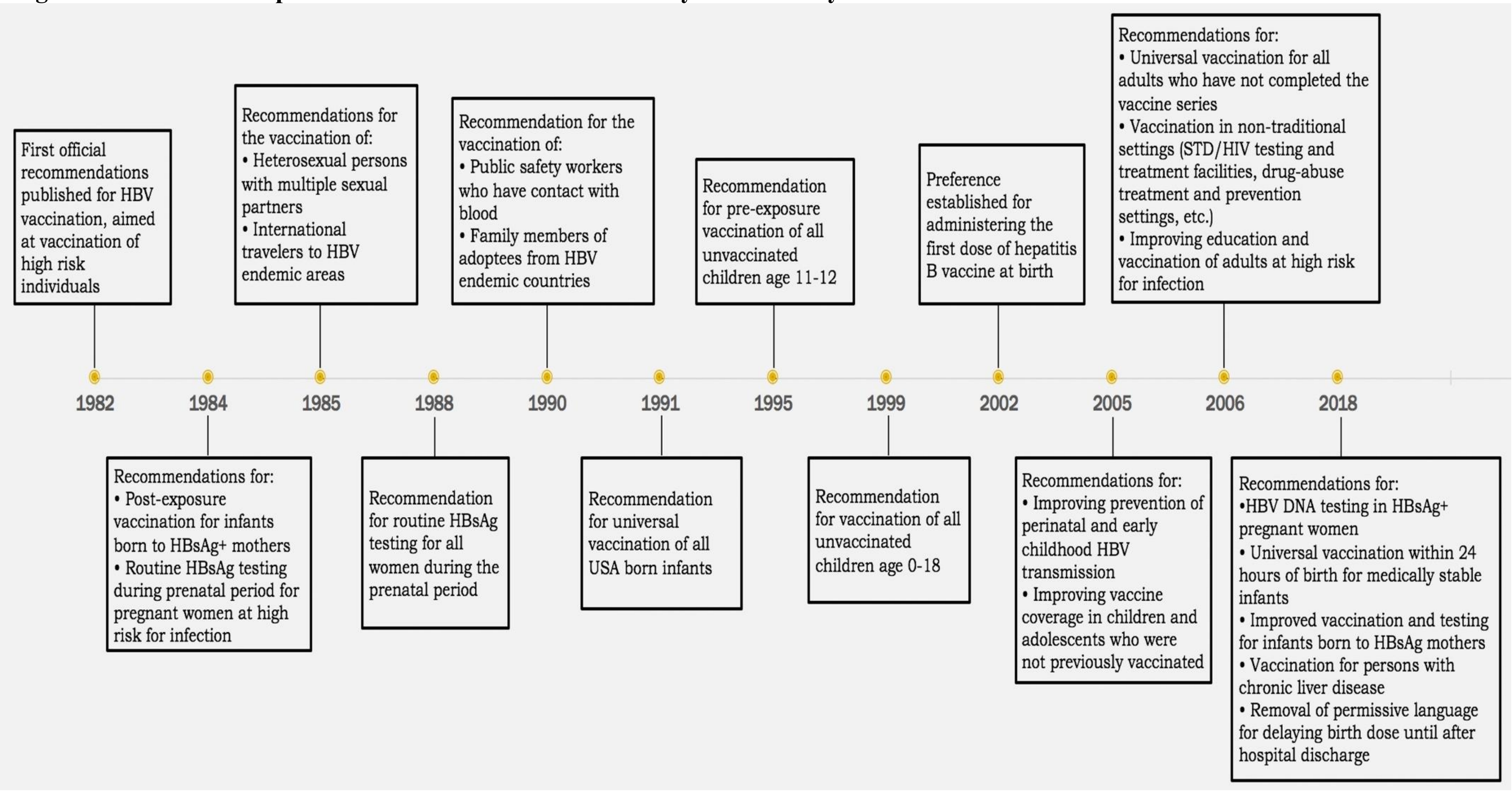


In 1991, the Advisory Committee on Immunization Practices (ACIP) recommended universal vaccination of infants for prevention of perinatal HBV transmission, as well as catch-up vaccinations for susceptible children and adult populations at high risk for infection In 2005, ACIP further solidified this recommendation by specifying that all newborns who weigh >2,000 g (4.4 lbs) and are medically stable receive their first dose of hepatitis B vaccine before discharge.

© 2020 Le MH et al. *JAMA Network Open.*

**eFigure 2. Timeline of hepatitis B vaccine availability in the United States**[13-21]

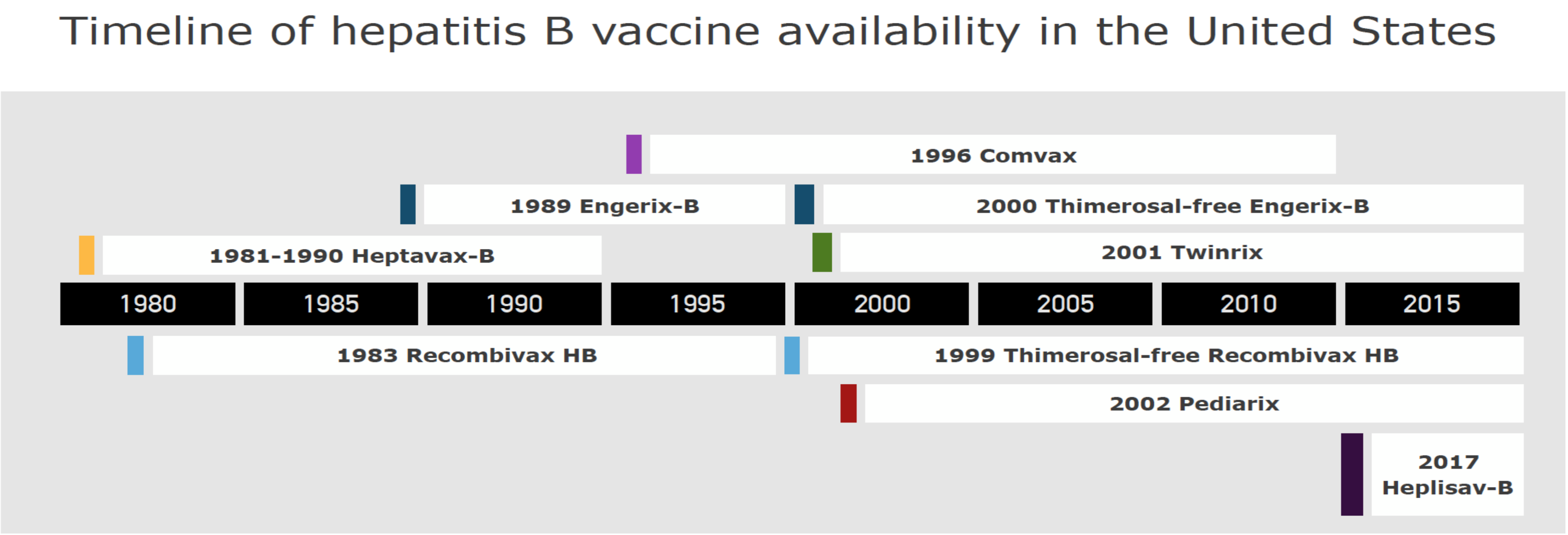


**Heptavax-B (Merck & Company, Inc.)**
Approved by the Food and Drug Administration (FDA) in 1981, Heptavax-B was the first hepatitis B vaccine and was prepared from the plasma of chronic HBV patients. Discontinued after 1990.

**Recombivax HB (Merck & Company, Inc.)**
Approved by the FDA in 1983 as the first recombinant hepatitis B vaccine. A thimerosal-free vaccine was approved by the FDA in 1999. Approved for use in individuals of all ages for the prevention of hepatitis B virus infection. Administered in a 3-dose schedule or 2-dose schedule for adolescents.

**Engerix-B (GlaxoSmithKline plc)**
Approved by the FDA in 1989. A thimerosal-free vaccine was approved by the FDA in 2000. Approved for use in individuals of all ages for the prevention of hepatitis B virus infection. Administered in a 3-dose schedule and 4-dose schedule for hemodialysis patients.

**Comvax (Merck & Company, Inc.)**
Approved by the FDA in 1996. Approved for use in individuals under 6 weeks of age for active immunization against invasive *Haemophilus influenzae* type B and HBV infection. Discontinued after 2014.

**Twinrix (GlaxoSmithKline plc)**
Approved by the FDA in 2001. Approved for use in subjects age 18 years or older. Indicated for use in active immunization against hepatitis A and hepatitis B. Administered in a series of 3-doses or 4-doses.

**Pediarix (GlaxoSmithKline plc)**
Approved by the FDA in 2002. Indicated for use in active immunization against diphtheria, tetanus, pertussis, poliomyelitis, and hepatitis B. Approved for use in subjects age 6 weeks to 6 years. Administered in a series of 3-doses.

**Heplisav-B (Dynavax)**
Approved by the FDA in 2017. Indicated for prevention of hepatitis B virus infection. Approved for use in adults age 18 years or older. Administered in a 2-dose series.

© 2020 Le MH et al. *JAMA Network Open.*

**eAppendix.Literature review on anamnestic response**

| **Study** | **No. of patients** | **Birth cohort** | **Country** | **Loss of immunity (%)** | **Booster vaccine and dose** | **Age at booster dose (year)** | **Anamnestic response** |
|---|---|---|---|---|---|---|---|
| Lin 2003[22] | 200 | **1987** | Taiwan | 71.1% at age 7, 37.4% at age 12 | Recombinant hepatitis B vaccines, 5 µg of Ricombivax [Merck] or 20 µg o | 7-12 | **60% after one year** |
| Boxall 2004[23] | 116 | **1986** | UK | 60% after 9 years follow-up | A dose, HB VAX II, Aventis Pasteur | (Mean) 11.7-14.5 | **83.6%** |
| Lu 2004[24] | 91 | **1988** | Taiwan | 48.7% after 15 years follow-up | A dose, 20 µg, recombinant DNA HB vaccine, Engerix-B | 15 | **89%** |
| Gabbuti 2007[25] | 20 | **1980 *(vaccinated in 1992)*** | Italy | 8.8% after 11 years follow-up | NA | 23 | **91.7%** |
| Jafarzadeh 2006[26] | 94 | **NA** | Iran | 52.1% after 10 years follow-up | NA | 10-11 | **95.8%** |
| van der Sande 2007[27] | 297 | **1986-1990** | Gambia | 66.5% after 15 years follow-up | A dose, Euvax® | 15 | **96.8%** |
| McMahon 2009[28] | 493 | **1981** | **US (Alaska)** | 40% after 22 years follow-up | A dose, 10 mg, Recombivax HB (Merck) | 22 | **81% at 60 days, 41% after one year** |
| Van Damme 2010[29] | 40 | **1986-1991 *(vaccinated in 2001)*** | Belgium, Australia | 79.5% in 2-dose group and 91.4% in the 3-dose group after 5 years follow-up | A dose, 10 µg, recombinant HBsAg, Engerix™-B | (Mean) 18.5 | **100%** |

© 2020 Le MH et al. *JAMA Network Open.*

**References**

1. CDC. Inactivated hepatitis B vaccine. MMWR 1982;31:317--8.
2. CDC. Postexposure prophylaxis of hepatitis B. MMWR 1984;33:285--90.
3. CDC. Recommendation of the Immunization Practices Advisory Committee (ACIP) Recommendations for Protection Against Viral Hepatitis. MMWR 1985;34:313-24,329-35.
4. CDC. Prevention of perinatal transmission of hepatitis B virus: prenatal screening of all pregnant women for hepatitis B surface antigen. MMWR 1988;37:341--6, 351.
5. CDC. Protection Against Viral Hepatitis Recommendations of the Immunization Practices Advisory Committee (ACIP). MMWR 1990;39(RR-2):1-26.
6. CDC. Hepatitis B virus: a comprehensive strategy for eliminating transmission in the United States through universal childhood vaccination. MMWR 1991;40(No. RR-13).
7. CDC. Update: recommendations to prevent hepatitis B virus transmission---United States. MMWR 1995;44:574--5.
8. CDC. Update: recommendations to prevent hepatitis B virus transmission---United States. MMWR 1999;48:33--4.
9. CDC. Notice to Readers: Recommended Childhood Immunization Schedule --- United States, 2002. MMWR 2002;51:31-3
10. Centers for Disease Control and Prevention. A comprehensive immunization strategy to eliminate transmission of hepatitis B virus infection in the United States: Recommendations of the Advisory Committee on Immunization Practices (ACIP); Part I: Immunization of Infants, Children, and Adolescents. MMWR 2005;54:1-3.
11. Centers for Disease Control and Prevention. A comprehensive immunization strategy to eliminate transmission of hepatitis B virus infection in the United States: Recommendations of the Advisory Committee on Immunization Practices (ACIP); Part II: Immunization of Adults. MMWR 2006;55:1-3.
12. Centers for Disease Control and Prevention. Prevention of Hepatitis B Virus Infection in the United States: Recommendations of the Advisory Committee on Immunization Practices. MMWR 2018; 67:1-2
13. Jr. SH. Vaccine for hepatitis B, judged highly effective, is approved by F.D.A. New York Times 1981 November 17.
14. Administration USFaD. Recombivax HB 1983 [Package Insert - Recombivax HB]. Available from: https://www.fda.gov/downloads/BiologicsBloodVaccines/Vaccines/ApprovedProducts/UCM110114.pdf.
15. Administration USFaD. ENGERIX-B 1989 [Package Insert - ENGERIX-B]. Available from: https://www.fda.gov/downloads/BiologicsBloodVaccines/Vaccines/ApprovedProducts/UCM224503.pdf.
16. Centers for Disease C, Prevention. Notice to Readers FDA Approval for Infants of a Haemophilus influenzae Type b Conjugate and Hepatitis B (Recombinant) Combined Vaccine. MMWR Morb Mrotal Wkly Rep 1997; 46(05):107-109.
17. Centers for Disease C, Prevention. Availability of hepatitis B vaccine that does not contain thimerosal as a preservative. MMWR Morb Mortal Wkly Rep 1999;48(35):780-2.
18. Centers for Disease C, Prevention. Update: expanded availability of thimerosal preservative-free hepatitis B vaccine. MMWR Morb Mortal Wkly Rep 2000;49(28):642, 51.


© 2020 Le MH et al. *JAMA Network Open.*

19. Administration USFaD. TWINRIX 2001 [Package Insert - TWINRIX]. Available from: https://www.fda.gov/downloads/BiologicsBloodVaccines/Vaccines/ApprovedProducts/UCM110079.pdf.
20. Administration USFaD. Pediarix 2002 [Package Insert - Pediarix]. Available from: https://www.fda.gov/downloads/BiologicsBloodVaccines/Vaccines/ApprovedProducts/UCM241874.pdf.
21. Administration USFaD. HEPLISAV-B 2017 [Package Insert - HEPLISAV-B]. Available from: https://www.fda.gov/downloads/BiologicsBloodVaccines/Vaccines/ApprovedProducts/UCM584762.pdf.
22. Lin YC, Chang MH, Ni YH, et al. Long-term immunogenicity and efficacy of universal hepatitis B virus vaccination in Taiwan. J Infect Dis 2003;187(1):134-8.
23. Boxall EH, J AS, El-Shuhkri N, et al. Long-term persistence of immunity to hepatitis B after vaccination during infancy in a country where endemicity is low. J Infect Dis 2004;190(7):1264-9.
24. Lu CY, Chiang BL, Chi WK, et al. Waning immunity to plasma-derived hepatitis B vaccine and the need for boosters 15 years after neonatal vaccination. Hepatology 2004;40(6):1415-20.
25. Gabbuti A, Romano L, Blanc P, et al. Long-term immunogenicity of hepatitis B vaccination in a cohort of Italian healthy adolescents. Vaccine 2007;25(16):3129-32.
26. Jafarzadeh A, Montazerifar SJ. Persistence of anti-HBs antibody and immunological memory in children vaccinated with hepatitis B vaccine at birth. J Ayub Med Coll Abbottabad 2006;18(4):4-9.
27. van der Sande MA, Waight PA, Mendy M, et al. Long-term protection against HBV chronic carriage of Gambian adolescents vaccinated in infancy and immune response in HBV booster trial in adolescence. PLoS One 2007;2(8):e753.
28. McMahon BJ, Dentinger CM, Bruden D, et al. Antibody levels and protection after hepatitis B vaccine: results of a 22-year follow-up study and response to a booster dose. J Infect Dis 2009;200(9):1390-6.
29. Van Damme P, Moiseeva A, Marichev I, et al. Five years follow-up following two or three doses of a hepatitis B vaccine in adolescents aged 11-15 years: a randomised controlled study. BMC Infect Dis 2010;10:357.

© 2020 Le MH et al. *JAMA Network Open.*
